# Factors contributing to the low uptake of medical male circumcision in Mutare Rural District, Zimbabwe

**DOI:** 10.4102/phcfm.v8i2.966

**Published:** 2016-05-31

**Authors:** Irene O. Chiringa, Dorah U. Ramathuba, Ntsieni S. Mashau

**Affiliations:** 1 Department of Public Health, University of Venda, South Africa

## Abstract

**Background:**

Medical male circumcision (MMC) has become a significant dimension of HIV prevention interventions, after the results of three randomised controlled trials in Uganda, South Africa and Kenya demonstrated that circumcision has a protective effect against contracting HIV of up to 60%. Following recommendations by the World Health Organization, Zimbabwe in 2009 adopted voluntary MMC as an additional HIV prevention strategy to the existing ABC behaviour change model.

**Purpose:**

The purpose of this study is thus to investigate the factors contributing to the low uptake of MMC.

**Methods:**

The study was a quantitative cross-sectional survey conducted in Mutare rural district, Zimbabwe. Questionnaires with open- and closed-ended questions were administered to the eligible respondents. The target population were male participants aged 15–29 who met the inclusion criteria. The households were systematically selected with a sample size of 234. Statistical Package for the Social Sciences was used to analyse the data.

**Results:**

Socioculturally, circumcised men are viewed as worthless (37%), shameful (30%) and are tainted as promiscuous (20%), psychological factors reported were infection and delayed healing (39%), being ashamed and dehumanised (58%), stigmatised and discriminated (40.2%) and fear of having an erection during treatment period (89.7%) whilst socio-economic factors were not having time, as it will take their time from work (58%) and complications may arise leading to spending money on treatment (84%).

**Conclusion:**

Knowledge deficits regarding male medical circumcision lead to low uptake, education on male medical circumcision and its benefits. Comprehensive sexual health education should target men and dispel negative attitudes related to the use of health services.

## Introduction

Male circumcision is one of the oldest and most common surgical procedures worldwide and is undertaken for many reasons, such as religious, cultural, social and medical. In Zimbabwe, male circumcision has been a practice amongst the social groups Shangani in Chiredzi and Mwenezi districts and the Varemba in Gutu and Mberengwa districts for traditional purposes. It has also been predominantly practiced by the Chewa and Muslim people for religious purposes.^1^

Zimbabwe has a projected population of 12.7 million people and is amongst the countries in Sub-Saharan Africa worst affected by the HIV and AIDS epidemic.^2^ The estimated HIV prevalence amongst adults 15 years and above in 2011 was 13.1% according to the National HIV Estimates of 2010. There were an estimated 1 159 097 adults and children who were living with HIV and AIDS in 2011. In the meantime, an estimated population of 597 293 adults and children were in urgent need of anti-retroviral therapy by the end of 2011. In the population (15 years and above), using the current 2010 EPP and Spectrum software, HIV prevalence in Zimbabwe was estimated to be 23.7% in 2001 and 18.4% in 2005 and further declined to 13.1% in 2011. The principal mode of HIV transmission in Zimbabwe is heterosexual contact, which accounts for 92% of all HIV infections in the country.^3^

In 2007, World Health Organization (WHO)^4^ and UNAIDS issued recommendations on medical male circumcision (MMC) as an additional HIV prevention strategy based on strong and consistent scientific evidence. Three randomised controlled trials undertaken in Kisumu, Kenya, Rakai District, Uganda and Orange Farm, South Africa, have shown that MMC reduces the risk of sexual transmission of HIV from women to men by approximately 60%. The most recent data from Uganda show that in the 5 years since the Uganda trial was completed, high effectiveness has been maintained amongst the men who were circumcised, with a 73% protective effect against HIV infection.

Furthermore, WHO^4^ recommended that the intervention should be added in countries with high HIV prevalence, generalised heterosexual HIV epidemics and low levels of male circumcision where the intervention is likely to have the greatest public health impact. Fourteen priority countries with this profile are striving to scale up voluntary MMC: Botswana, Ethiopia, Kenya, Lesotho, Malawi, Mozambique, Namibia, Rwanda, South Africa, Swaziland, Tanzania, Uganda, Zambia and Zimbabwe.

Following the recommendations of the WHO, Zimbabwe introduced the male circumcision programme in November 2009 as an additional HIV prevention strategy to the existing ABC behaviour change model. According to the Ministry of Health,^5^ the Zimbabwean government plans to circumcise about 3 million men by the end of 2015, but the target may be overly ambitious, as less than 10% of the targeted population has yet been circumcised. Zimbabwe carries the third largest burden of HIV cases in Southern Africa after South Africa, with the largest burden globally of 5.6 million people living with HIV in 2010 followed by Mozambique with 1.4 million people in 2010.^6^ There is need, therefore, to scale up efforts in providing prevention interventions to reduce the occurrence of new infections.

## Purpose of study

To investigate the factors contributing to the low uptake of MMC in Mutare rural district.

### Significance of the study

The results provided the nation with information with regards to why men in rural areas are reluctant to engage and partake in this programme despite its protective effect. The information acquired is of use to the National AIDS Council (NAC) and the Ministry of Health and Child Welfare in helping to develop an awareness programme. Furthermore, the results of the study provide baseline information that will assist health planners to design effective strategies directed towards dealing with factors that are hindering uptake of MMC in rural areas.

### Methodology

The study used a descriptive cross-sectional survey, which describes a phenomenon at one point in time. Its focus is to observe, describe and document aspects of a situation as it naturally occurs.^7^ Description of the responses of participants in proportions, frequencies or percentages assisted in bringing out the factors contributing to the low uptake of MMC. The target population were all male participants aged 15–29 in Bambazonke Village, which has four sections. Sampling is a process of taking a portion or a smaller number of units of a population as representative or having particular characteristics of that total population.^8^ Systematic sampling was used to select households in this study. The sample size was proportionally divided according to the population of each section in the village. A self-administered questionnaire was used for collecting data. The questionnaire had four segments: biographical data, socio-economic factors, sociocultural factors and psychological factors contributing to the low uptake of MMC. Pretesting of the research instrument was done in Zvipiripiri, a village in Mutare rural district prior to actual data collection from 25 randomly selected male participants aged 15–29 years. Statistical Package for the Social Sciences software, version 17 was used to analyse the data.

### Ethical considerations

According to Babbie and Mouton,^9^ ethics in research refers to a general obligation for researchers to conduct their craft in a socially responsive and responsible manner. Permission to conduct the study was obtained from the University of Venda Research committee (Project no: SHS/14/PH/16/0312) and local leaders of Mutare rural district and the respective ward and village leaders (head men and councillors). Participants were informed about the purpose of the study. Informed consent was obtained and consent forms were signed. Confidentiality was assured and the right to autonomy and self-determination were taken into consideration.

## Results

The findings are presented and discussed according to the following sections: demographic information, sociocultural, psychological and socio-economic factors leading to the low uptake of MMC (Table 1). The findings are presented as descriptive summaries in the form of frequencies and percentages, where appropriate cross-tabulations between responses and respondent characteristics are presented

**TABLE 1:** Biographic data.

Variable	*N*	%
**Age of respondent, years**		
18–29	129	55
30–40	84	36
41–49	19	9
**Level of education**		
Grade 1–7	111	52
Secondary education	52	25
No formal education	50	24
**Employment status**		
Employed	103	44
Unemployed	131	56
**Marital status**		
Married	105	45
Single	70	30
Divorced	33	14
Widow	26	11
**Religion**		
Christian	129	55
Islam	96	41
Baha I’ Faith	7	3

*Source*: Authors’ own work

### Information on circumcision

Findings revealed that 18 (17%) were circumcised, and 216 (83%) were not circumcised. The reasons given were reduced risks of STIs 48%, reduces HIV infection (41%), and minority (6%) gives status in society, 8% sexual pleasure and 5% for religious purpose. For the uncircumcised, reasons given were unsatisfactory sexual performance 70 (30%), fear of pain 63 (27%), ancestors’ permission 44 (19%), being shunned by the community 42 (18%) and fear of the unknown 16 (7%).

Furthermore, 61 (26%) indicated removal of foreskin, 70 (30%) removal of head of the penis, 68 (29%) preparation for manhood and 5 (15%) had no idea.

### Personal views regarding type, place and age of circumcision

### Sociocultural factors

Decision making regarding circumcision was made by fathers in 95 (40.5%) respondents, 86 (36.7%) made the decision themselves, 42 (17.9%) took the help of extended family members and 8 (3.4%) indicated grandparents to have made the decision. Regarding their views, 87 (37%) reported that circumcision is viewed as worthless, 30% as shameful, 20% attached it with promiscuity, 23 (10%) viewed it as honourable, whilst 3% felt it is defied by the gods.

### Psychological factors

Of the respondents, 71% feared surgical operation, pain, bleeding and other complications; 74% had fear of infection and delayed wound healing; 145 (62%) felt circumcision dehumanises; 54 (23%) were not sure and 10% did not feel ashamed or dehumanised. Regarding stigma and discrimination, 159 (68%) felt it would lead to stigma, 26 (11%) had no fear of stigma and discrimination whilst 21% indicated it no impact on them. Participants were in disagreement (150 [64%]) that circumcision gives a false sense of security, 73 (31%) were unsure and 9 (4%) agreed that it does. Concerning HIV testing, prior circumcision was considered as an obstacle by 121 (51.7%) whilst 72 (30.7%) were not certain and 48 (20.5%) felt that it was not an obstacle. Pertaining to women’s preferences of circumcised men, 157 (67%) disagreed, 19 (8%) agreed and 59 (25%) were not sure. The majority (168 [72%]) reported that ‘it reduces penis size whilst did not agree, 136 (58%) indicated fear of losing a partner during wound healing time, whilst 98 (42%) did not’. Ninety-five percent reported fear of loss of erection as compared to 12 (5%) and 204 (87%), who agreed that it diminished sexual pleasure whilst 30 (13%) disagreed. Regarding preferences of women with circumcised men, 154 (66%) disagreed, 70 (30%) agreed and 9 (4%) were not sure

### Socio-economic factors

One hundred and sixty-eight (72%) agreed to not having time, 28 (12%) disagreed and 37 (16%) were not sure. Furthermore, 211 (95%) reported absence from work whilst 5% said it was not an issue and 115 (49%) were uncertain if persistent pain may result in job loss; 69 (29%) disagreed and 12 (5%) agreed. Regarding transport money to health service, 211 (95%) disagreed and only 9 (4%) agreed that it is a barrier.

## Discussions

Circumcision is a sociocultural issue and needs concerted effort in improving its uptake as majority of participants held different views that prevented them from using circumcision services. When curbing the scourge of HIV, adolescents are the most targeted group and older men are excluded from prevention strategies. The findings indicated that the age category 18–29 years had the highest rate of participants with 129 (55%), followed by the middle aged at 84 (36%) and lastly the 41- to 49-year category at 21 (9%). The analysis by the Ministry of Health and Child Welfare and the NAC^10^ indicated that ‘24.6% of the entire adult population aged 15–49 is currently infected, making Zimbabwe one of the most seriously affected countries in the entire world’. It is important that preventive strategies target men above 30 years because men are not using health services and engage in risky sexual behaviours, as condom use is usually promoted amongst adolescents and not amongst men in unions. Nearly half (122 [52%]) of the respondents have attained basic education. The education level of participants is amongst the important characteristics as it is associated with many factors that have a significant impact on health-seeking behaviours; however, this is not the case. Maja^11^ indicated that males had limited knowledge regarding contraceptives and were not consistent in condom use to prevent unwanted pregnancies and suggested male empowerment and inclusion in reproductive health services.

The high rate of unemployment could be attributed to the fact that in this study most respondents are in the age category 18–29 and more likely to be currently unemployed than their counterparts in older age groups as they are still pursuing their education. Religious and cultural beliefs were observed as barriers contributing to low uptake; this is asserted by the fact that Zimbabwe is mainly constituted of people with traditional beliefs and Christians as reflected by the views of 129 (55%) and 96 (41%) respondents, respectively. The Islum, baha’I faith constitutes a small fraction of the population. Religious affiliation has an influence on one’s decision to undergo circumcision or not. According to Salem,^12^ circumcision is one of the oldest operations in history within Jewish religion; male infants are traditionally circumcised on their eighth day of life, provided there is no medical contraindication. The justification behind this is that a covenant was made between Abraham and God.^13^ However, Christians retain many of the features of early Christianity, of not opting for male circumcision based on the scripture by St Paul in (Galatians 5:6): ‘in Christ Jesus neither circumcision nor uncircumcision count for anything’.^12^

Other personal and ethnic factors can hinder the decision of male circumcision. Table 2 reflects that those who had undergone circumcision had done so traditionally (138 [59%]); only 49 (21%) had the procedure done medically. This is also in correlation with the religious affiliation of the study sample as most of them reflected that they had traditional beliefs as per their religion. In conjunction with the above findings, ‘other studies conducted amongst different ethnic groups in Africa have found that male circumcision is carried out for traditional reasons, as an initiation ritual and a rite of passage from childhood into manhood’.^14,15^ Therefore, this indicates how tradition and/or culture has an influence on MMC. Regarding the best age for circumcision, most of the respondents viewed at birth as the most convenient. ‘The findings by Tarimo^16^ corroborate with the present study as they postulate that in their study men preferred circumcision at early childhood because young boys express less pain than adult men and reasoned that men have matured vessels which may cause more pain to them’. Decision making to circumcise depends on the general views of the community or values of cultures and subcultures. A qualitative study by Mavhu et al.^17^ indicates that fathers are decision-makers in the acceptability of male circumcision on their infants, where uncircumcision is acceptable. Ogbonnaya^18^ reported that women had concerns about the sudden interest of their partners to undergo circumcision, insinuating that they are having sex with other women. This concurs with the study findings that circumcised men are viewed as promiscuous.

**TABLE 2:** Type of circumcision undertaken and the best place and age for circumcision.

Type of circumcision	*N*	%	Best place for circumcision	%	Best age for circumcision	*N*	%
Medical	49	21	Clinic/hospital	16	New born baby	87	37
Traditional	138	59	Home	10	2–6 years	5	12
Religious	33	14	Traditional setting	24	7–13 years	9	4
None of the above	14	6	Church	20	Above 20 years	56	24
			Not acceptable	30	Unsure	55	23

*Source*: Authors’ own work

Almost three-quarters of the population defined circumcision wrongly, some indicated that they did not know what it is, while others viewed it as removal of the penis head and had sociocultural perceptions that circumcision is a sinful act and that nobody has the power to change what God has created. Bailey^19^ concurs with the above as he asserted low acceptability of male circumcision amongst Christians because of the belief that it was a sin to change the way one was created. Furthermore a study by Kelly et al.^20^ shares the same view as that mentioned above, as amongst Christians in Papua New Guinea, male circumcision was considered unacceptable because they believed that HIV prevention was found in God from being unfaithful.

In addition, statistics have shown low uptake of MMC in rural areas than in urban areas, which coincides with the present study. A comparison of the reasons for getting circumcised and not getting circumcised was made. The findings reflect that those who are circumcised had knowledge on the benefits that circumcision has as most of them stated it reduces HIV transmission by 60% and that it reduces the risk of HIV infection. This is supported by a study finding of Mhangara,^21^ which affirms that knowledge of the benefits of male circumcision is paramount in building a positive perception of the procedure.

Those who had not been circumcised opined that circumcision will lead to unsatisfactory sexual performance and pain and thus preferred to avoid it. Hargreave^22^ enunciates the same concern that both male and female sexual enjoyment is compromised by MMC. The results of the present study do not show a relationship between educational level and the definition of circumcision. Most studies reveal that education level has an impact on knowledge. The results of the study by Mbusa and Nkala^23^ also showcased a lack of in-depth knowledge about the benefits and limitations of MMC and without knowledge people are reluctant and sceptical about it. Furthermore, 183 (78%) respondents indicated they were not circumcised, leaving nearly a quarter of them saying they were circumcised. This study concurs with the study by Fritz et al.^24^ in terms of the high rate of uncircumcision, which was 201 (86%).

Participants had other psychological factors related to anxiety about fear of surgical operations; pain, bleeding and other complications are the reasons for not undergoing MMC as reflected in Table 3, with only 49 (20%) participants in disagreement. Similar findings were reflected on the factors associated with uptake of voluntary MMC in Mazowe District Zimbabwe by Rapfute^25^ reflecting fear of pain and possibility of complications such as death as what was preventing men for undergoing MMC. Fear of death may have been stimulated by national media reports that confirmed 5 deaths of adolescent boys because of inept and unhygienic ritual circumcision by some cultural groups in South Africa in June 2001.^26^ However, Darby^27^ states that ‘death from MMC is exceedingly rare since they are mostly done under general anesthesia and local anesthesia’. There is a possibility that wound healing might be delayed during male circumcision especially when there is an infection. Furthermore, Westercamp and Bailey^28^ in a study conducted in Kenya identified the perception of long healing period following circumcision procedure as a barrier to circumcision.

**TABLE 3:** Levels of agreement on psychological factors.

Psychological factors	Level of agreement (%)				

	SD	D	U	A	SA
1. I fear surgical operation, pain, bleeding and other complications	9.4	10.7	9.0	46.6	24.4
2. I fear that circumcision would lead to infection and wound will take too long to heal	9.4	8.5	7.7	35.5	38.9
3. I feel ashamed and dehumanised due to circumcision	3.0	6.8	22.6	9.4	58.1
4. I fear being stigmatised and discriminated	6.0	4.7	20.9	28.2	40.2
5. Circumcision gives me a false sense of security	1.3	63.2	31.2	0	4.3
6. HIV testing before the procedure prevents me from get circumcised	13.7	6.8	30.8	9.0	39.7
7. I heard women do not like circumcised men	38.5	27.8	25.2	5.1	3.4
8. Circumcision reduces penis size	17.1	10.3	0	52.5	20.1
9. I fear losing my partner or wife during the waiting period	32.9	8.5	0	47.0	11.5
10. I may lose the capability of having an erection and I am scared of having an erection during waiting period	5.6	0	0	4.7	89.7
11. Sexual pleasure is diminished when a person is circumcised and I might end up losing my partner	2.1	11.1	0	85.1	1.7
12. Women prefer to have sex with men who are circumcised	29.5	36.8	4.3	18.8	10.7

*Source*: Authors’ own work

Similar findings were noted by Herman-Roloff,^29^ which revealed that delayed wound healing and prolonged time away from work are common barriers of male circumcision uptake amongst men. It is therefore paramount that the authorities responsible improve the quality of MMC services focusing on reducing delayed wound healing time so that this does not stand as a barrier towards MMC. Only a tenth of the population indicated that circumcision did not make them feel ashamed or dehumanised, 67% of them stated that MMC would bring feelings of shame and make them feel dehumanised, and this is supported by Plotkin,^30^ ‘who acknowledged that older men would lose face if they met younger men or boys in this settings’. Furthermore, ‘their status within the family and society would be demeaned if they get circumcised at the perceived inappropriate age of circumcision’. A minority (26 [11%]) of the respondents in the study had no fear of stigma and discrimination. Mbusa^23^ asserts that ‘depending on the cultural values and norms of a particular community, men and boys undergo circumcision for fear of being stigmatised especially if the majorities are circumcised’. The study findings revealed that nearly half of the respondents [115 (49%)] regarded HIV testing before the procedure as the reason for not wanting to be circumcised. Gwata^31^ coincides with the above assertion that ‘fear of HIV results made some people to dread going for circumcision as they know it is a prerequisite before circumcision’.

Regarding the notion that women do not like circumcised men, 157 (67%) of the respondents were in disagreement with it. Only 8% were in agreement with the notion that women do not like circumcised men, and 59 (25%) stated that they were unsure about women’s preference. Above half of the respondents shared sentiments of fear of losing a partner during waiting period, whilst 98 (42%) did not share the same sentiments. Khehla^32^ shares the same view ‘as respondents hesitated waiting for six weeks for the wound to heal before resuming sexual activity’. The results of the study indicated fear of loss of capability of having an erection after circumcision as well as having an erection during waiting period as a major barrier for circumcision as reflected by 222 (95%) respondents. Plotkins^30^ states that fear of penile injury from erections in the immediate post-operative period also emerged as a potential barrier. Fink^33^ also ‘reported worsened erectile function after adult circumcision and, in addition, a degradation of penile sensitivity’. A different school of thought argues that MMC leads to prolongation of ejaculation and latency time, which may be an added advantage in young men in whom quick ejaculation is very frequent. Noel^34^ concluded overall satisfaction rates despite slightly reduced erectile function and decreased penile sensitivity.

Majority (204 [87%]) of the respondents were in agreement that circumcision diminished sexual pleasure and this would lead them to lose their partner. Thirteen percent of the respondents did not think it would diminish their sexual pleasure or lead them to losing their partner. Wilcken^35^ opposes the above assertion as he postulates that younger men felt circumcision enhanced sexual pleasure and satisfaction for women, a belief that is widely held amongst both young men and women. Pang^36^ carried out a survey in South Korea and reported that a man is twice as likely to experience diminished sexuality rather than improved sexuality. Furthermore, findings reflected that most women prefer to have sex with uncircumcised men 154 (66%). Studies done by various authors indicated that women were significantly more likely to report vaginal dryness with a circumcised partner.^37^ Socio-economic factors also contributes to the uptake because most of the respondents (222 [95%]) stated circumcision as likely to take their time away from work. According to Sengwayo,^38^ ‘it is a deterrent as most men want to keep it private and personal’.

### Recommendations

Data analysed indicated reluctance by older men to engage in MMC as compared to younger men. Therefore, in order for MMC implementers and health planners to boost MMC uptake, more resources should be spent on circumcising younger men and more campaigns in schools and engaging MMC in their school curricula would be of more influence and older men should also be targeted.

Culture and religion were shown to have a great impact on decision making regarding MMC. Dissemination of information should be increased in the various religious and traditional groups as they are holding back on circumcision due to certain misconceptions. Leaders of these groups should be engaged as they have a great influence.

Misconceptions and myths on MMC remain high especially in rural areas as indicated in the study; therefore, provision of accurate information is of paramount importance in order to educate people on the benefits and risks associated with MMC. The Ministry of Health and Child Welfare should come up with more campaign strategies in order to increase the adoption of MMC.

The Ministry of Health and Child Welfare should come up with strategies to curb the following major barriers identified in the study: long healing time, pain, complications such as death, costs incurred before and after circumcision and also providing accurate information on MMC.

### Limitations

The methodological limitation of this study is that it used a small sample from Mutare District. Therefore, the results may not be generalised to the entire population, including men in other districts of Zimbabwe.

## Conclusion

The survey indicates the need to improve the uptake of male circumcision so as to decrease the burden of AIDS in Sub-Saharan Africa in line with reducing the mortality and morbidity of HIV and/or AIDS. The health belief theory suggests that perceived susceptibility influenced change in behaviour. Intensive health education campaigns on the benefits of male circumcision, inclusion in the curricula, and a multi sectoral approach with community leaders and private sector to improve acceptability are required.
